# Mesoscale insights in Epileptic Networks: A Multimodal Intracranial Dataset

**DOI:** 10.1038/s41597-025-05026-4

**Published:** 2025-05-10

**Authors:** Vasiliki Bougou, Michaël Vanhoyland, Evy Cleeren, Peter Janssen, Wim Van Paesschen, Tom Theys

**Affiliations:** 1 Research Group of Experimental Neurosurgery and Neuroanatomy, Department of Neurosciences, KU Leuven and the Leuven Brain Institute, Leuven, Belgium; 2 Laboratory for Neuro – and Psychophysiology, Research Group Neurophysiology, Department of Neurosciences, KU Leuven and the Leuven Brain Institute, Leuven, Belgium; 3 Department of Neurosurgery, University Hospitals Leuven, Leuven, Belgium; 4 Department of Neurology, University Hospitals Leuven, Leuven, Belgium; 5 Laboratory for Epilepsy Research, KU Leuven, Leuven, Belgium

**Keywords:** Epilepsy, Epilepsy

## Abstract

Understanding the intricate dynamics of epileptic networks at the mesoscale is crucial for advancing our knowledge of epilepsy pathophysiology and developing targeted interventions. In this data descriptor, we present a comprehensive dataset encompassing intracranial electroencephalography (iEEG) recordings, Local Field Potentials (LFP), and Multiunit Activity (MUA) data obtained from Microelectrode arrays (MEA; Utah array; Blackrock). 12 seizures were recorded in 5 epilepsy patients with the MEA. Our dataset offers a unique opportunity to investigate the complex interactions between diverse neural signals across brain areas and to study the mesoscale networks in focal epilepsy. This dataset can be used to explore the modulations of LFP and MUA in conjunction with iEEG, offering potential insights into the spatiotemporal dynamics of epileptic networks. Additionally, the high temporal resolution of the data allows for the computation of High-Frequency Oscillations (HFOs) in both LFP and iEEG signals, facilitating the investigation of their potential relationship with MUA activity.

## Background & Summary

Epilepsy, which is characterized by recurrent and unpredictable seizures, affects millions of individuals worldwide. A significant proportion of cases are resistant to pharmacological treatment, and a similar fraction presents significant side effects to anti-seizure medication (ASM)^1,2^. Understanding the neural dynamics underlying epileptic seizures is pivotal for improving patient care, treatment options, and seizure prediction.

A common approach for refractory epilepsy involves identifying the Seizure Onset Zone (SOZ) using intracranial EEG (iEEG). Intracranial EEG recordings offer a unique window into the brain’s electrical activity, allowing to investigate the spatiotemporal patterns and dynamics associated with seizures. Recently, High-frequency oscillations (HFOs) have emerged as crucial markers of epileptic activity,with both interictal and ictal HFOs providing valuable insights into epileptogenic networks and contributing to the identification of the seizure onset zone^3–8^. Notably, in epilepsy surgery, the removal of tissue exhibiting HFOs appears to predict favorable surgical outcomes, often outperforming the removal of the ictal onset zone^9^. This highlights the significance of considering HFOs in clinical evaluations. However, the underlying pathophysiology of epileptic HFOs remains largely unclear. Previous studies have established a strong correlation between high-gamma activity and the firing rates of neurons^10^. Consequently, it becomes imperative to study HFOs at a microscopic level to unravel their origin and pathophysiological mechanisms.

While recent advances have been made in epilepsy research, the development of reliable mechanisms for seizure prevention and prediction remains a challenge. Traditional research has primarily focused on macro-scale analyses, necessitating a broader understanding of epileptic networks at the mesoscale level. This entails the exploration of multi-unit activity (MUA) and local field potentials (LFP) recorded by microelectrode arrays (MEAs). Schevon *et al*.^11^ utilized high-resolution microelectrode arrays to examine electrocortical activity in the epileptogenic cortex, revealing that both interictal and ictal discharges occur in very small cortical regions, as small as 200 μm². This work underscores the critical role of these localized discharges in understanding seizure initiation and propagation. In a subsequent study, Schevon *et al*.^12^ identified significant contrasts in neuronal firing patterns between core seizure regions and surrounding areas, highlighting a distinct ‘ictal penumbra’ where local field potentials may be large even if neuronal firing rates remain modest. Other studies have also reported that during clinical seizures, intense synchronized neuronal activity is often not observed, with increased firing rates found in only a small fraction of units^12–17^. Schevon *et al*.^18^ hypothesized that the heterogeneous firing patterns observed in some microelectrode recordings might reflect the penumbra rather than the core ictal region, suggesting that variability in firing patterns could be indicative of regions surrounding the active seizure focus. Additionally, high spatial resolution recordings have proven useful for seizure prediction, as demonstrated by Truccolo *et al*.^19^, who reported significant alterations in spiking activity even beyond the seizure onset zone (SOZ) minutes before a seizure. The combination of LFP and MUA data from microelectrode arrays situated outside the SOZ has also shown promise in the reliable detection of epileptic seizures^20^. This body of evidence highlights the importance of high spatial resolution recordings for accurately capturing seizure dynamics and improving prediction and intervention strategies.

We introduce a comprehensive dataset encompassing iEEG, MUA, and LFP recordings obtained from epilepsy patients during seizures. The dataset encompasses cases with identified presumed epileptogenic zones and those with a less distinct focus, offering a spectrum of epilepsy dynamics. It provides a unique opportunity to merge data from multiple spatial scales and investigate their behavior and interactions before, during, and after seizures. The consistent proximity of Utah arrays to macroelectrode contacts enables intricate explorations of the relationship between distinct neural signals. Additionally, the dataset offers varying distances of MEAs from the SOZ, facilitating comparisons of MUA and LFP activity and their interactions with iEEG across different Utah array locations in relation to the SOZ. Lastly, this dataset could provide insights into the pathophysiology of ictal HFOs by analyzing their detection in Local Field Potentials (LFPs) recorded through Multi-Electrode Array (MEA) analysis, alongside observing spiking activity from the same channels. Overall, this dataset serves as a valuable resource to enhance our understanding of meso- and macro-epileptic networks, contributing to advancements in seizure prediction and pathophysiology.

## Methods

Data were collected from five patients (patient 1, 23-y-old man; patient 2, 60-y-old woman; patient 3, 26-y-old man; patient 4, 31-y-old man; patient 5, 57-y-old woman) with intracranial depth electrodes as part of their presurgical evaluation for drug resistant focal epilepsy. The implantation and recording procedures for patients 2 and 5 have been previously described in^21^ while those for patients 2 and 4 were detailed in^22^. Ethical approval was obtained for the implantation of microelectrode arrays (Utah arrays) in patients with epilepsy as part of a clinical trial (study number s53126). The placement of other electrodes (depth electrodes, ECoG grids, and high-density ECoG grids) followed standard clinical care protocols. Study protocol s53126 was approved by the local ethical committee (Ethische Commissie Onderzoek UZ/KU Leuven) and was conducted in compliance with the principles of the Declaration of Helsinki, the principles of good clinical practice, and in accordance with all applicable regulatory requirements. All human data were encrypted and stored at the University Hospitals Leuven. All patients provided informed consent, including explicit permission for the processing and sharing of encrypted study data for research purposes and its publication within the context of the study.

### Patients

Five patients underwent surgical implantation of depth electrodes, electrocorticography (ECoG) grids, and/or high density electrocorticography grids as part of their epilepsy surgery workup. In addition to clinical electrodes, patients were implanted with microelectrode arrays (Utah arrays) for research purposes, with the aim of investigating the mesoscale dynamics of the epileptic network. The arrays were only implanted if a craniotomy was performed for the placement of subdural grids or strips, therefore, the implantation of the arrays did not lead to additional incisions. The arrays were placed in close proximity to the subdural grids to study the microscale dynamics of the epileptic network. This was clearly discussed with all patients during the preoperative consultation (approximately 1-2 months before surgery) and the day before surgery. Arrays were inserted in or near the presumed epileptogenic zone (based on preoperative multimodal imaging). Therefore, the brain tissue at the implantation site was a potential resection site prior to the recordings. After analysis of the intracranial EEG, it was deemed that the array was not inserted in the actual epilepticogenic zone (in patient 1 and 2 a remote focal onset zone was detected, patient 3 had multifocal epilepsy). Importantly, none of the patients has experienced complications related to the micro-electrode array. After two weeks (14–16 days) the arrays were removed together with the other clinical intracranial electrodes in a second surgery.

**Fig. 1:**
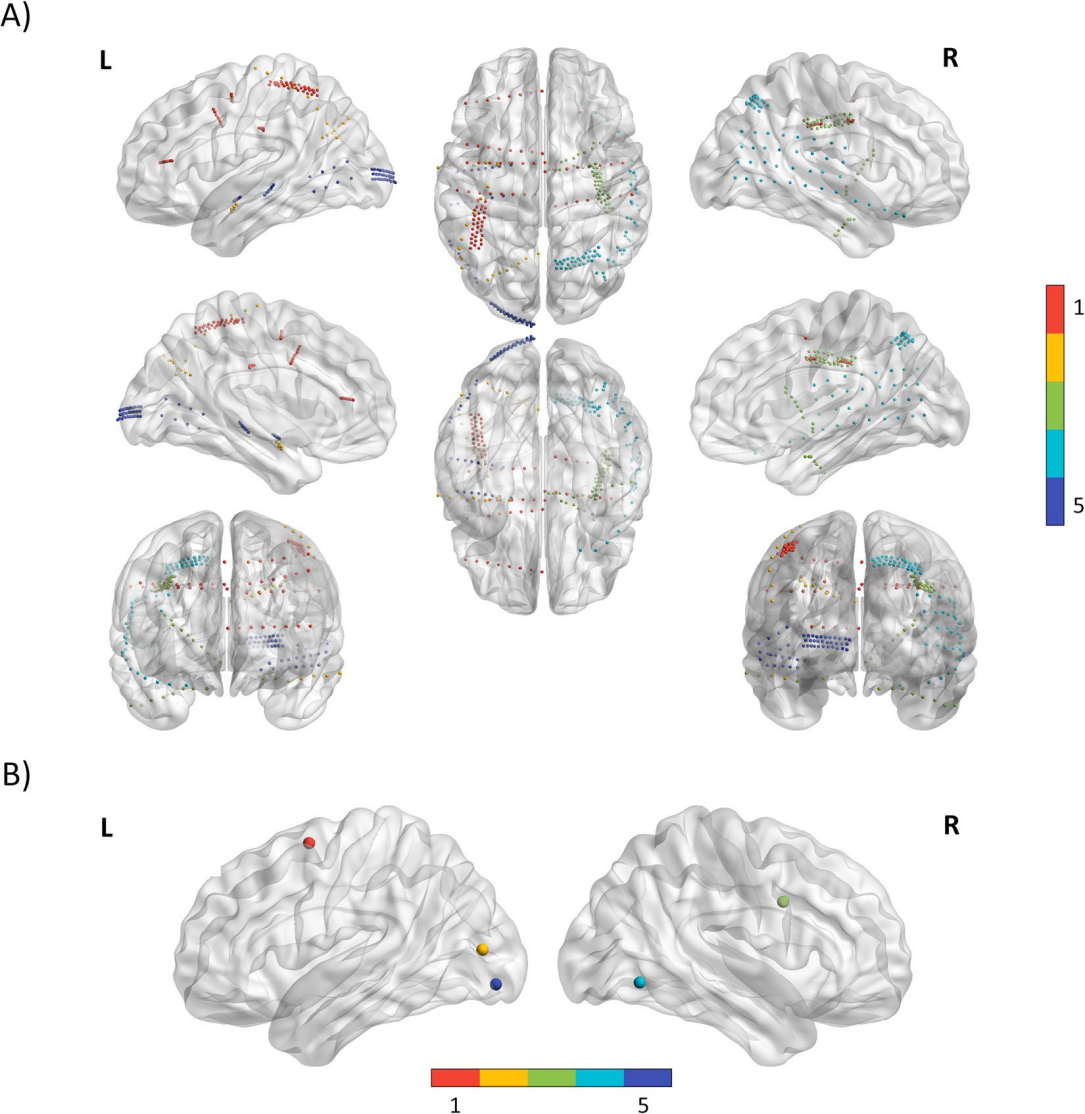
(**A**) SEEG and ECoG electrodes from all patients were combined and reconstructed on an MNI brain. Electrodes are depicted as small dots or markers. The five different colors correspond to the five different patients. (**B**) Utah arrays from all patients are plotted as dots on one common MNI brain. The five different colors correspond to the five different patients.

The area and number of channels of implanted electrodes for each patient are detailed in Table 1, while the technical details of the sEEG, ECoG grids, and strips are described in Tables 2, 3, respectively. Importantly, no additional incisions were made for the study. The three Utah arrays (patients 2, 4, and 5) were located in the occipitotemporal cortex (OTC), while the remaining two (patients 1, and 3) were in the dorsolateral prefrontal cortex (PFC) adjacent to clinical electrodes. The target locations for intracranial electrode placement were determined by the multidisciplinary epilepsy surgery team, including epileptologist and neurosurgeon, guided by electroclinical findings and non-invasive multimodal imaging techniques. Figure 1 shows the reconstruction of the SEEG, ECoG electrodes, and the Utah arrays for all patients. The electrodes were visualized with the BrainNet Viewer (http://www.nitrc.org/projects/bnv/)^23^.

**Table 1:** Electrodes per patient.

Patient 1	Patient 2	Patient 3	Patient 4	Patient 5
**A:** 8 – channel depth electrode (Type 1) in left anterior cingulate cortex	**A:** 8 – channel depth electrode (Type 1) in left amygdala	**A**: 10 – channel depth electrode (Type 2) in hypothalamic hamartoma	**A:** 4 × 8 grid (Type 3) over previous resection area (right parietal)	**A:** 32 – channel grid (Type 1) over left occipital cortex
**B:** 8 – channel depth electrode (Type 1) in left anterior cingulate cortex	**B:** 8 – channel depth electrode (Type 1) in left mesial occipital cortex	**B:** 8 – channel depth electrode (Type 1) in amygdala	**B:** 6 – channel strip (Type 4) over right anterior temporal cortex	**B:** 10 – channel depth electrode (Type 2) in left amygdala
**C:** 8 – channel depth electrode (Type 1) in left supplementary motor area	**C:** 8 – channel strip (Type 2) over left occipital cortex	**C:** 32 – channel grid (Type 1)	**C:** 32 – channel grid (Type 1) over right parietal area	**C:** 10 – channel depth electrode (Type 2) in left hippocampus
**D:** 8 – channel depth electrode (Type 1) in left anterior cingulate cortex	**Utah:** 96 channel Utah array in occipitotemporal cortex	**Utah:** 96 channel Utah array in prefrontal cortex	**Utah:** 96 channel Utah array in occipitotemporal cortex	**D:** 2 × 4 grid (Type 5) over venous infarction on the left posterior temporal lobe
**E:** 32 – channel grid (Type 1)				**Utah:** 96 channel Utah array in occipitotemporal cortex
**F:** 8 – channel depth electrode (Type 1) in right anterior cingulate cortex				
**G:** 8 – channel depth electrode (Type 1) in right posterior cingulate cortex				
**Utah:** 96 channel Utah array in dorsal prefrontal cortex

**Table 2:** Technical details SEEG electrodes.

Type	Company	Contacts	Diameter	Contact length	Intercontact distance
Type 1	PMT Corp.	8	0.8 mm	2 mm	7 mm
Type 2	PMT Corp.	10	0.8 mm	2 mm	3.5 mm

**Table 3:** Technical details ECoG.

Type	Company	Contacts	Diameter	Intercontact distance
Type 1	PMT Corp.	32 (3 × 11, 11-10-11)	1 mm	3 mm
Type 2	PMT Corp.	8 (1 × 8)	3 mm	10 mm
Type 3	PMT Corp.	32 (4 × 8)	3 mm	10 mm
Type 4	PMT Corp.	6 (1 × 6)	3 mm	10 mm
Type 5	PMT Corp.	8 (2 × 4)	3 mm	10 mm

### Clinical information

**Patient 1** experienced epilepsy since childhood, characterized by frequent nocturnal seizures with a frontal lobe semiology. Structural MRI showed no focal lesions. Seizures evolved from focal to bilateral tonic-clonic (FTBC) events with strong ictal activity on grid contacts located on the secondary somatosensory cortex (S2). One focal impaired awareness seizure (FIAS), characterized by the same hyperkinetic semiology as the FBTC seizures, lacked a clear EEG correlate, and cortical stimulations failed to elicit seizures. Therefore, the precise onset location could not be determined.

**Patient 2** suffered from focal seizures with impaired awareness, presumably a postlesional epilepsy after a craniocerebral trauma at age 11. In 2011, the patient experienced a second craniocerebral trauma, leading to a right-sided epidural hematoma. MRI showed the temporal post-traumatic lesion with parenchymal loss, secondary gliosis and ex-vacuo dilatation of the temporal horn of the left lateral ventricle. While seizure semiology, MRI findings, and interictal EEG supported left temporal lobe epilepsy, ictal EEG and SISCOM suggested a left posterior ictal onset zone. Invasive EEG revealed different seizure onset zones, including temporal neocortex, mesial occipital cortex and few seizures from which the onset was not sampled with invasive EEG as the first ictal signals were recorded on the scalp EEG. Therefore, the epileptogenic zone could not be reliably determined.

**Patient 3** experienced focal aware seizures with a history of two radiosurgical treatments for a hypothalamic hamartoma. Aside from the irradiated hypothalamus/tuber cinereum region, the MRI was normal. Seizures were consistent with right temporal lobe epilepsy. Interictal EEG findings revealed IEDs primarily at contact points in the amygdala and temporal neocortex, coinciding with scalp EEG. The seizure onset zone was not encompassing the hamartoma. A left temporal lobe resection, excluding the hippocampus, was performed. Postoperatively, the patient experienced frequent minor seizures, with a notable absence of major seizures.

**Patient 4** experienced focal seizures that progressed to bilateral tonic-clonic seizures. Structural MRI showed no focal lesions. In 2012, the patient underwent resective epilepsy surgery at the right temporoparietal junction but continued to experience inadequate seizure control. Post-operative MRI showed gliosis at the borders of the resection cavity. Intracranial EEG indicated that seizures originated from the anterior border of the previous resection, and an additional resection was performed. While initially seizure-free for several months, the patient later experienced recurrent seizures.

**Patient 5** experienced seizures after a left parieto-occipital intracerebral hematoma resulting from venous sinus thrombosis. Structural MRI showed left hippocampal sclerosis in addition to the left posterior inferior and middle temporal old venous infarct with porencephaly, surrounding gliosis and encephalomalacia. Seizures were focal with impaired awareness and started in the hippocampus before spreading to the amygdala. Surgical intervention involved a left temporal lobe resection with amygdalohippocampectomy and resulted in a favourable outcome.

### Data acquisition

Patients were implanted with platinum-iridium depth or SEEG electrodes. Each electrode shaft contained between 8 and 10 electrode contacts. All patients, except patient 2, were implanted with 32-channel high-density grids (PMT, size 39 × 12 mm, diameter of each channel 1 mm, with an inter-contact distance of 3 mm). Patients 2, 4, and 5 were also implanted with platinum subdural ECoG grids, each containing between 6 and 32 contacts. All contacts were connected to a 128- channel neural signal processor (BrainRT^TM^ EEG equipment).

Additionally, 96 – channel microelectrode arrays (4 × 4 mm; electrode spacing of 400 microns; Blackrock Microsystems, UT) were inserted in all patients. The arrays were placed using a pneumatic inserter wand (Blackrock Neurotech) with a single hit (20 psi). Reference wires were placed in the subdural spaces, ground wires epidurally. The dura was closed above the array and the bone flap was placed on top to secure the array and prevent array flotation. Reference wires were placed subdurally and ground wires epidurally. The signal was digitally amplified by a Cereplex M headstage (Blackrock Neurotech) and recorded with a 128 – channel neural signal processor (NeuroPort system, Blackrock Neurotech, Salt Lake City, UT, USA). In each recording session, multi – unit activity (MUA) from all 96 channels was sampled at 30 kHz, and high-pass filtered above 750 Hz. The detection trigger of the MUA was set at the edge of the noise band. The LFP signals were recorded continuously with a sampling frequency of 1000 Hz.

To minimize noise, the clinical recording system and the micro array neural signal processor were plugged in different outlets (so not on the same distribution plug) and were grounded to separate ground plugs within the room. All electronics (including bed, tv, cellphone chargers) were unplugged from the outlets to reduced 50 Hz line noise during daytime recordings but were left plugged in during sleep recordings. For the intracranial EEG, a macro-contact within white matter (based on CT-MRI coregistration) was used as reference electrode, while an electrode on the mastoid was used for grounding.

Patients were monitored for a total of 2 weeks (14–16 days). During this period, patients were daily evaluated for signs of neurological deficit by one of the neurosurgeons. None of the patients suffered from a neurological deficit/bleeding/infection in the period after implantation of the intracranial electrodes.

Impedance data from the manufacturer was provided on a separate CD and verified prior to implantation. Impedance checks were not conducted in the operating room before implantation to minimize the risk of infection. Starting from patient 2, impedance measurements were performed regularly during the monitoring period. These checks were not conducted systematically but occurred approximately 3–4 times throughout the recording period.

### Annotation of the intracranial EEG

The SEEG was continuously monitored in the Epilepsy Monitoring Unit and annotated for interictal and ictal activity. Clinical SEEG annotation was performed by a senior EEG expert with more than 5 years of experience in intracranial and scalp EEG analysis and one epileptologist with more than 15 years of clinical experience. If both readers would disagree the seizure onset and termination times of a particular seizure, it was discussed during the multidisciplinary team meeting consisting of different experts regarding epilepsy surgery. Preoperative clinical information and patient demographics are provided in Table 4. Further information on seizure onset sites, ictal EEG onset patterns and interictal discharge populations are summarized in Table 5.

**Table 4:** Surgical information.

	Gender	Age	Presurgical evaluation	Post implantation surgical procedure	Engel	ILAE	Pathology report	Time since iEEG (months)
Patient 1	M	19	iiEEG: midfronto – central icEEG: midfronto – central semiology: frontal MRI: negative PET: left mesiofrontal SISCOM: left/midline mesiofrontal MEG: left operculum, inferior frontal and premotor	No resection	No resection	No resection	No resection	61
Patient 2	F	58	iiEEG: left temporo – frontoparietal icEEG: left centroparietal semiology: left temporal MRI: left temporal posttraumatic lesion PET: left temporal and posterior SISCOM: left posterior temporoparietal	No resection	No resection	No resection	No resection	32
Patient 3	M	24	iiEEG: right temporal icEEG: right temporal semiology: right temporal MRI: hypothalamic harmatoma PET: right temporal SISCOM: right temporal	Left temporal lobe resection, excluding the hippocampus. Array location was not resected.	IVB	5	Hippocampus showing neuronal loss, consistent with hippocampal sclerosis.	46
Patient 4	M	29	iiEEG: right temporoparietal icEEG: right temporoparietal MRI: right temporoparietal gliosis at old resection cavity PET: right temporoparietal SISCOM: right temporoparietal	Resection at right temporoparietal junction. Array location was not resected.	IIIA	4	Mild nonspecific changes with gliosis; no evidence of focal cortical dysplasia.	31
Patient 5	F	55	iiEEG: bitemporal icEEG: left temporal semiology: left temporal MRI: left hippocampal sclerosis and left posterior temporal venous infarct PET: left temporal SISCOM: left inferolateral temporal	Left temporal lobe resection with amygdalo – hippocampectomy. Array location was not resected.	IB	2	Hippocampus showing neuronal loss, consistent with hippocampal sclerosis.	36

Pre-operative information for all patients, including demographic details (gender, age) and results from presurgical evaluations (iiEEG: interictal electroencephalography; icEEG: ictal electroencephalography; MRI: magnetic resonance imaging; PET: positron emission tomography; SISCOM: Subtraction Ictal SPECT coregistered to MRI; MEG: magnetoencephalography). Engel and ILAE classifications indicate surgical resection status and time since the initial intracranial EEG (iEEG) in months.

**Table 5:** Post – implantation clinical information.

	# of seizures	# of seizures recorded with Utah array	Days post – implantation for Utah array seizures	Seizure types	Channels with interictal DIscharges	Other Notable Findings	Seizure onset sites	Onset patterns
Patient 1	11	6	4-5-9-12-13-13	10 FBTC; 1 short FIAS (same focal semiology as the FBTC, no EEG correlate)	Spikes: E2-3-4-5-12-13-14-15-23-24-25-26; Repetitive Spikes: C5		Unknown; first ictal activity on E (anatomical location S2)	LVFA on E5-8, 15-18,26-30
Patient 2	80	1	15	79 subclinical; 1 FIAS	Spikes: B7-9; Repetitive Spikes: B2, B3		B1-2-3 or B 6-7-8 (65 seizures); A5-6 (6 seizures); scalp EEG (8 seizures)	79 subclinical: ○ LVFA B1-2-3 or B6-78(65 seizures) ○ LVFA A5-6 (6 seizures) 1 FIAS: LVFA B1-2-3
Patient 3	114	2	13	FIAS (majority preceded by an aura)	NSpikes: B2-6; Low Voltage Spikes: A1	Sharp Waves: C3, C14, C15	B2-3; B1-2 (amygdala and temporal neocortex)	LVFA B2-3; Repetitive spiking theta range B2-3; LVFA B1-2; Global attenuation iEEG and scalp EEG followed by: ○ LVFA B4-5 ○ LVFA A and B2-3 ○ widespread beta,mostly in B4-5 ○ LVFA A and B4-5
Patient 4	1	1	9	FBTC	Spikes: A2-4, A12	Beta Activity: A23-32	A4	LVFA A4
Patient 5	56	2	2	50 subclinical; 6 FIAS	Spikes: C1-3; Concomitant Spikes: B1-3		C1-2-3	LVFA or repetitive spiking evolving to LVFA

Post-operative clinical information for all patients, including the total number of seizures recorded, the number of seizures captured with the Utah array, and the days post-implantation for the array. The table also details seizure types (FBTC: focal to bilateral tonic-clonic seizures; FIAS: focal impaired awareness seizures), channels with interictal discharges, seizure onset sites, and onset patterns. Interictal discharges are categorized as spikes (sudden, sharp waveforms indicating brief, high-voltage electrical discharges), repetitive spikes (a series of spikes occurring in succession), low voltage spikes (spikes characterized by lower amplitude), and concomitant spikes (spikes occurring simultaneously in different channels). In the onset patterns column, LVFA denotes low voltage fast activity.

### Anatomical labelling

To perform the electrode localization of the depth electrodes, subdural electrodes, and Utah arrays, first, a preoperative T1-weighted MRI scan and a postoperative computed tomography (CT) scan were resliced and coregistered using SPM software (https://www.fil.ion.ucl.ac.uk/spm/). Then, the scans were parcellated using Freesurfer (http://surfer.nmr.mgh.harvard.edu/), and lastly, the electrodes were visualized and localized using the iElectrodes^24^ MATLAB toolbox.

### Experimental design

Seizures for Patients 1, 2, and 5 occurred during nocturnal sleep, while Patients 3 and 4 experienced seizures during experimental tasks conducted for separate studies. Patient 3 engaged in grasping imagery, while Patient 4 participated in a flash suppression visual task. To ensure data synchronization, a photodiode was fixed to the left upper corner of the task-displaying screen, detecting a white square that coincided with the start of each trial. The photodiode signal was simultaneously recorded by both the signal processors of the intracranial EEG and the microelectrode array, facilitating alignment between datasets. During nocturnal recordings, the intracranial EEG data were continuously recorded throughout the night. Utah array data were segmented into one-hour files, with the activation of a photodiode signal at the start of each segment, ensuring synchronization with the intracranial EEG neural processor recordings.

### Data pre – processing

The intracranial EEG data were sampled at 1024 Hz, except for Seizures 1 and 2 in Patient 1, which were sampled at 256 Hz. LFP data were consistently sampled at 1000 Hz, and MUA data were downsampled to 1000 Hz as well. Data analysis was conducted using custom MATLAB R2020b scripts (MathWorks, Natick, MA, USA) and the EEGLAB toolbox^25^. To eliminate line noise and artifacts while preserving non-artifact components, we applied a combined spectral and spatial filter^26^. Subsequently, a zero-phase Finite Impulse Response (FIR) high-pass filter with a cut-off frequency above 3 Hz was employed. Temporal alignment of the data was performed based on seizure onset annotations by the epileptologist. In most cases, the epoch consisted of 10 minutes before the seizure and 5 minutes after. However, adjustments were necessary in specific instances because some seizures did not occur during continuous overnight recordings but while the patients were performing experiments. In these instances, we started the recording when the experiment began. If a seizure occurred before 10 minutes of pre-seizure data could be recorded, the epoch was adjusted to the maximum available pre-seizure recording time. Patient 3’s seizures included data from 8 minutes before and the second seizure analysis included data from 2 minutes after seizure onset, Patient 4’s first seizure included 4 minutes before and 5 minutes after, and Patient 5’s second seizure analysis included data from 4 minutes after seizure onset. These seizures did not occur during continuous overnight recordings but while the patients were performing experiments. In these instances, we started the recording when the experiment began. If a seizure occurred before we had 10 minutes of recording from its onset, the epoch had to be adjusted to the maximum time we managed to record before the seizure.

## Data Records

The dataset^27^ is available at EBRAINS repository:

The structure is the following:

**repository-root/**

*<***sub - X>/** *[data specific for subject X]*

*<***ieeg_filtered>/** *[intracranial pre-processed neural data]*

**Sub-X-seizure-Y-ieeg.mat** *[structure containing pre – processed SEEG data of patient X for seizure Y (data), sampling Frequency (Fs), low and high frequency of bandpass filtering (Flow, and Fhigh respectively), channel labels (labels), sample of the seizure onset (seizureSample)]*

**Sub-X-seizure-Y-lfp.mat** *[structure containing pre – processed Utah array LFP data of patient X for seizure Y (data), sampling Frequency (Fs), low and high frequency of bandpass filtering (Flow, and Fhigh respectively), channel labels (labels), sample of the seizure onset (seizureSample)]*

**Sub-X-seizure-Y-mua.mat** *[structure containing downsampled Utah array MUA data of patient X for seizure Y (data), sampling Frequency (Fs), channel labels (labels), sample of the seizure onset (seizureSample)]*

**<ieeg_raw>/** *[intracranial raw neural data]*

**Sub-X-seizure-Y-ieeg.edf** *[raw SEEG data of patient X for seizure Y around the defined epoch]*

**Sub-X-seizure-Y-lfp.mat** *[matrix (channels x time) containing raw (Fs = 1 kHz) Utah array LFP data of patient X for seizure Y around the defined epoch]*

**Sub-X-seizure-Y-mua.mat** *[matrix (channels x time) containing raw (Fs = 30 kHz) Utah array MUA data of patient X for seizure Y]*

*<***anat>/** *[imaging data]***<preop_MRI_3D_T1 >** *[Preoperative 3D T1 weighted MRI scan in DICOM format]***<postop_CT >** *[Postoperative CT scan in DICOM format]*

**Fig. 2:**
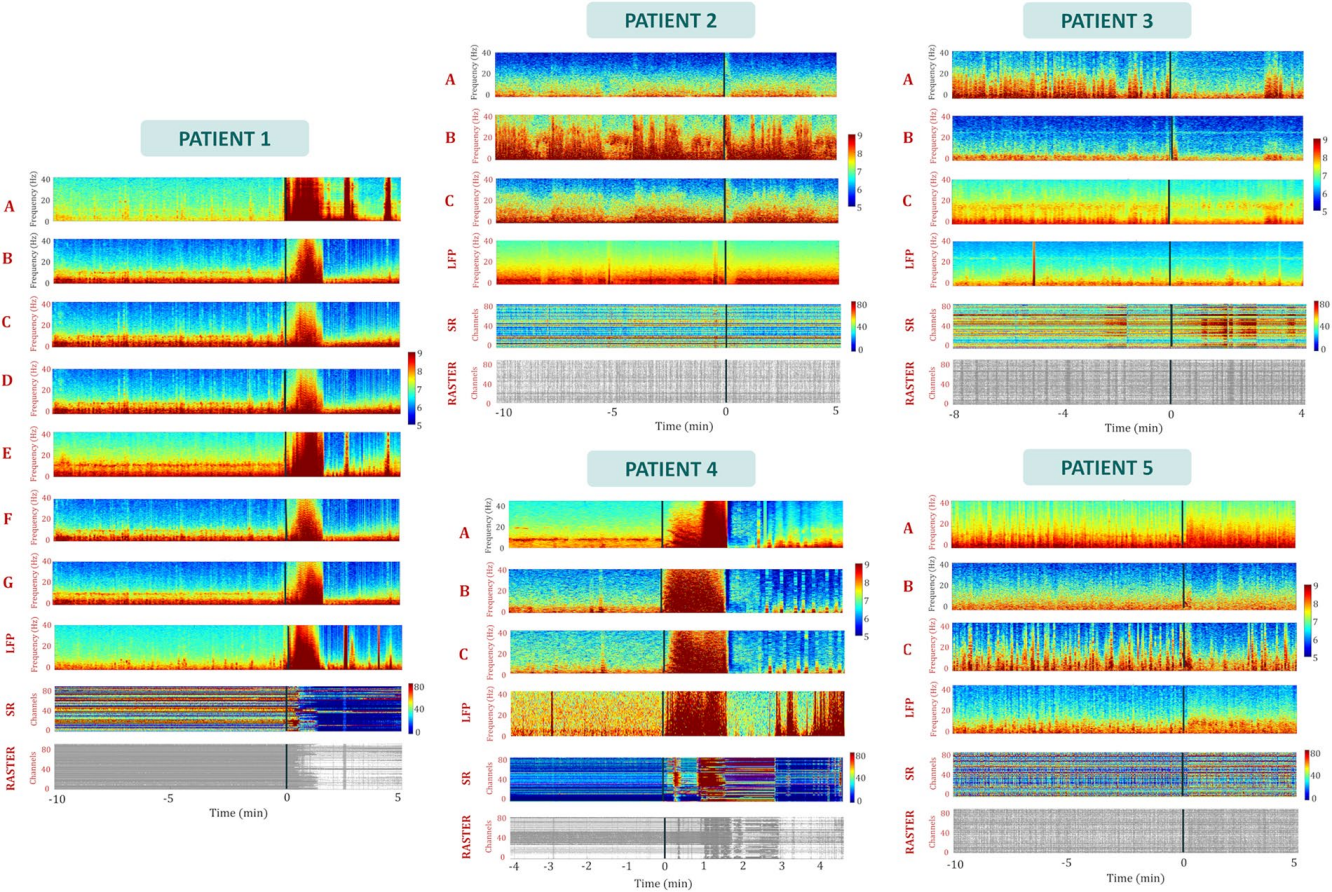
Power spectra and spike rate plots for all invasive electrodes. An example contact from each electrode and grid is displayed for all patients. Additionally, the power spectrum of the Local Field Potential (LFP) recorded by the Utah array is presented, averaged across all 96 channels. The color-coded spike rate is plotted for all channels, accompanied by spike data represented as a raster plot for each channel. The vertical axis denotes time with zero indicating seizure onset. Power values are represented on a logarithmic scale, and frequencies span from 2 to 40 Hz.

**mni_coordinates.txt** *[labels and X,Y,Z MNI coordinates of all electrodes] ***<impedances.txt >** *[impedance measurements for all electrodes and for different days post implantation]*** < layout_impedances.xlsx >** *[manufacturing impedance measurements for all electrodes and pinout diagrams for each array]*** < Matlab > /** *[Matlab script] ***<plot_data.m > ***[Matlab script that computes and displays the plots in* *Figure 2]***<Instructions.docx > ***[Instructions to run the script]*

## Technical Validation

Several steps were taken to validate our dataset. Time-frequency decomposition was performed, and power spectra were plotted for all seizures to analyse their spectral characteristics. Additionally, we plotted the broadband signal from one representative electrode, which showed the most epileptic activity per patient, alongside the average LFP across the array for a straightforward comparison of the two modalities. We evaluated the Euclidean distance and correlation between the two. Furthermore, we investigated the presence of High Frequency Oscillations (HFOs) in both intracranial EEG and Utah array Local LFP data and associated their presence with the Seizure Onset Zone (SOZ). We also visualized MUA patterns and identified significant modulations. These modulations were categorized based on their temporal alignment with seizures to assess their predictive value and consistency across seizures. Principal Component Analysis (PCA) was performed to the MUA data to explore potential patterns before or after seizure onset. Lastly, the spike – local field potential (LFP) correlation for different time epochs around the seizure was investigated.

### Time – frequency analysis

We performed spectral analysis using a Fast Fourier Transformation (FFT) on the iEEG and LFP pre – processed data. Power was computed within 1 sec windows, with a 50% overlap between consecutive windows. This allowed us to capture changes in the spectral content over time with high temporal resolution.

### Euclidean distance

For each patient, we selected one representative iEEG channel that exhibited the most epileptic activity during seizure onset, as determined by the epileptologists (patient 3: channel B2, patient 4: channel A4, patient 5: channel C1). In cases where multiple channels showed epileptic activity, we chose one (patient 1: channel E7, patient 2: channel B7). The Euclidean distance between the MNI coordinates of the Utah array and the corresponding electrode for each patient was calculated using the following formula:

$$d=\sqrt{{({x}_{1}-{x}_{2})}^{2}+{({y}_{1}-{y}_{2})}^{2}+{({z}_{1}-{z}_{2})}^{2}}$$

Where:

- *d* is the Euclidean distance.

- ${x}_{1},{y}_{1},{z}_{1}$ are the coordinates of the first point (e.g., the Utah array).

- ${x}_{2},{y}_{2},{z}_{2}$ are the coordinates of the second point (e.g., the electrode).

### Pearson correlation

For each patient, we identified the channels exhibiting the most epileptic activity in the iEEG as described above (patient 1: channel E7, patient 2: channel B7, patient 3: channel B2, patient 4: channel A4, patient 5: channel C1). Additionally, we determined the iEEG channel with the smallest Euclidean distance from the Utah array for each patient (patient 1: channel C5, patient 2: channel C8, patient 3: channel C11, patient 4: channel A3, patient 5: channel A32). For these channels, as well as for the average LFP signal across the Utah arrays, we extracted the broadband signal from an epoch spanning 0.5 seconds before the seizure and 1.5 minutes after the seizure onset. We used these time segments to compute the Pearson correlation coefficient for the following signal combinations: most epileptic channel – Utah array, nearest electrode – Utah array, and most epileptic channel – nearest electrode.

### High frequency oscillations (HFOs)

We employed a MATLAB graphical user interface^28^ to detect ictal High-Frequency Oscillations (HFOs) in both intracranial EEG (iEEG) and Local Field Potential (LFP) data. This toolbox offers an automated detection feature, which we initially utilized, followed by a visual inspection to manually eliminate any false positives. We began by re-referencing the data using a bipolar reference to the nearest contact, a common practice in HFO analysis. Subsequently, we opted for the Hilbert detector^29^ to identify ictal HFOs. This method involves initial signal filtering within a user-defined frequency range, followed by envelope computation using the Hilbert transform. To qualify as a valid event, two criteria were established: first, the local maximum for each event had to exceed a threshold of 5 standard deviations (SD) of the envelope, which was initially calculated over the entire recording or a specified time interval. Second, each detected ictal HFO was required to have a minimum duration of 10 milliseconds. For our analysis, we filtered the data within the 80 to 250 Hz frequency range to target HFOs.

### Multiunit activity (MUA)

For the Multiunit Activity (MUA) data initially, we extracted digitized spike events using the original sampling frequency of 30 kHz. This was done based on a threshold set during recording, approximately −3 standard deviations from the noise. Then, we detected timepoints in milliseconds corresponding to spike events and created a matrix with ones and zeros (sampled at 1 kHz for 1 ms precision). Lastly, we calculated the spike rate in 1-second windows. To improve data visualization in a raster plot, we further downsampled by retaining one sample every 20 milliseconds, resulting in a final sampling frequency (Fs) of 50 Hz. This downsampling was essential for enhancing the clarity of modulations in the plot.

### Detection of significant MUA modulations

To detect significant spike rate modulations, for each channel, we calculated the slope and identified values that exceeded three times the standard deviation (STD) of the entire time series. By considering time points where significant modulations were detected across all channels, we determined the time point with the highest likelihood of a significant modulation. Subsequently, we organized the time points corresponding to significant modulations and calculated the percentage within seven distinct groups, ranging from −10 minutes before the seizure to 4 minutes after, with a 2-minute interval.

### Evaluating consistency across seizures

For patients with more than one recorded seizure (patients 1, 3, and 5), we aimed to determine whether the channels displaying significant MUA modulations (slope exceed 3*STD of the entire time series) demonstrated consistency across different seizures. To achieve this, we employed the Jaccard coefficient, a quantitative measure widely used in set theory and statistics to assess the similarity between two sets. The Jaccard coefficient quantifies the similarity between two sets by calculating the size of their intersection divided by the size of their union. In our study, each set represented channels that displayed significant MUA modulations during different seizures, categorized within specific time groups (defined across a time window spanning from −10 minutes before seizure onset to 4 minutes after, with a 2-minute interval.). By applying the Jaccard coefficient, we evaluated the degree of overlap between sets, revealing the extent to which channels with significant MUA modulations during one seizure for a specific time group were consistent with those of another seizure.

### Principal components analysis (PCA)

We performed PCA on the spike rates recorded across channels and visualized the temporal patterns of the first three principal components.

### Spike – local field potential (LFP) correlation

We used a similar approach as described in Schevon *et al*.^12^. We evaluated the phase locking of spiking activity to the LFP for three 5-second intervals: 5 minutes before the seizure (‘pre’), immediately after seizure onset (‘early’), and 2 minutes after seizure onset (‘late’). For these segments and for all channels, we plotted the timing of the MUA circularly versus the instantaneous phase of the LFP of the same channel for different frequency bands. Instead of analyzing only the broadband low-frequency signals (2–45 Hz), we also analyzed narrower frequency bands: theta (4–8 Hz), alpha (8–12 Hz), beta (13–30 Hz), and low gamma (35–45 Hz), since the phase is more accurately estimated for narrowband signals that resemble an actual sinusoid. In contrast, broadband signals’ phase tends to be noisier due to the presence of multiple sinusoidal waves of different frequencies. To compute the instantaneous phase, we used a 4th-order IIR Butterworth bandpass filter to isolate the frequencies of interest and then applied the Hilbert transform to extract the instantaneous phase. We performed this analysis for all channels and for the three time segments. We then binned the MUA activity into 1 ms bins to downsample it, ensuring the same sampling frequency for both the LFP and the MUA. Next, we detected the spike timings for each channel and plotted them circularly versus the instantaneous phase of the LFP signal at the corresponding spiking times. We computed the correlation between spiking timing and LFP phase using the Rayleigh Z statistic. We calculated the significance using a bootstrap procedure, in which the MUA activity was circularly shifted relative to the LFP by a random time interval. The 95th percentile of the Rayleigh Z statistic for 10,000 trials was used as the threshold of significance. Lastly, for all patients and each of the three time intervals, we computed the number of channels showing significant phase locking.

## Results

### Different modulations across seizure types

**Fig. 3:**
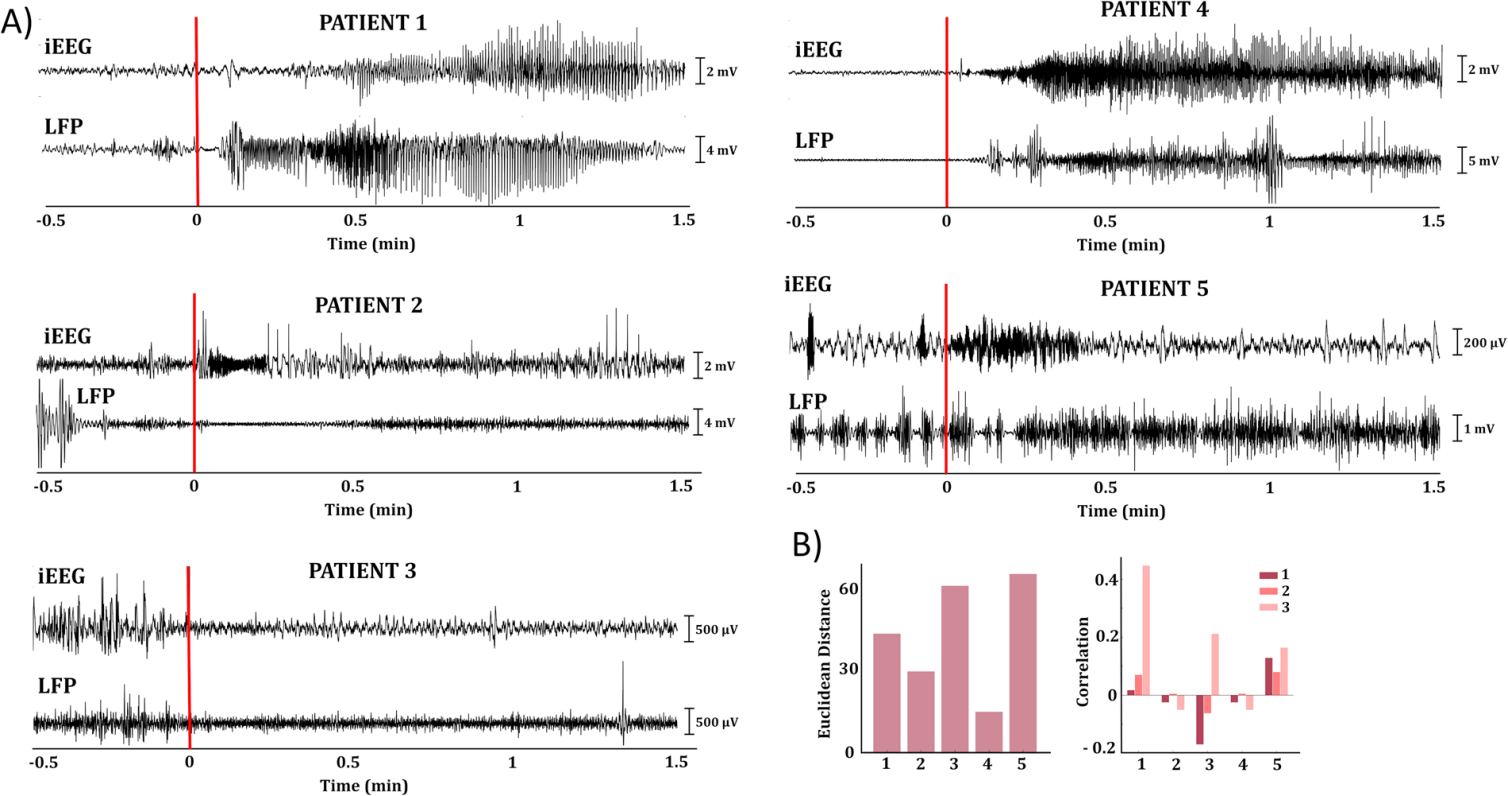
Broadband signals, Euclidean distances and correlation between contacts around seizure onset. (**A**) The broadband signal is plotted for an epoch spanning 0.5 minutes before seizure onset to 1.5 minutes after seizure onset for one contact per patient, reported by clinicians as either the contact with the most epileptic activity or one of multiple contacts. The selected contacts are: patient 1: E7, patient 2: B7, patient 3: B2, patient 4: A4, patient 5: C1. The average LFP across the array is also shown for each patient for the same time epoch. The vertical axis represents time, with zero indicating seizure onset. The bars within each plot indicate the peak-to-peak voltage. (**B**) Left: Bar plot showing the Euclidean distances between the MNI coordinates of the iEEG contacts with the most epileptic activity and the Utah array for each patient. Right: Bar plot displaying the Pearson correlation coefficients for each patient between three signals, epoched around 0.5 minutes before and 1.5 minutes after seizure onset: 1) the signal of the iEEG contact with the epileptic activity (same as in A), 2) the LFP of the Utah array, and 3) the signal of the iEEG contact located closest to the Utah array.

We plotted the power spectra for the intracranial EEG (iEEG), Local Field Potential (LFP), and the raster plot of the downsampled Multiunit Activity (MUA) and evaluated the modulations occurring before and after seizure onset (Fig. 2). Patients with a well-defined seizure focus (Patients 2, 3, 5) exhibited distinct spectral patterns, particularly noticeable between 3 and 20 Hz, in the electrode showing the most epileptic activity. Importantly, these patterns were evident throughout the entire 10-minute epoch preceding the seizure, rather than at seizure onset. Interestingly, no conspicuous modulations in spike rate, Local Field Potential (LFP), or intracranial EEG (iEEG) were observed following seizure onset in these cases. In contrast, patients with a less clearly defined focus (Patients 1, 4) and pronounced seizure propagation showed a broadband increase in power immediately after seizure onset across all iEEG channels. Prior to the seizure onset, the power spectrum exhibited similar characteristics across all channels. Additionally, these patients exhibited robust broadband power increases in the LFP signal, with simultaneous onsets. Notably, strong Multiunit Activity (MUA) modulations were also observed. Specifically, for Patient 1, there was a noticeable spike rate inhibition approximately 1 minute after seizure onset, while for Patient 4, an increased spike rate emerged between 1 and 3 minutes after seizure onset, followed by inhibition. When examining the plots of the raw broadband signal (Fig. 3A), we observe a similar trend. Patients 1 and 4 display almost simultaneous, clear onsets in both the iEEG and the Utah array LFP, while the results for the other patients are more heterogeneous. Patient 3 exhibits suppression in both the iEEG and LFP signals immediately after seizure onset. In patients 2 and 5, although heightened activity following seizure onset is captured in the iEEG contact, the LFP activity appears suppressed for patient 2. In contrast, patient 5’s LFP shows a rhythmic pattern before and during the early stages of the seizure, evolving into increased activity approximately 15 seconds after seizure onset. The differences observed across patients suggest that seizure dynamics, including pre-seizure modulations and post-onset changes, may vary significantly depending on individual factors such as seizure focus, brain region, and network properties. This variability is indicative of the non-uniform nature of epileptic network responses, potentially driven by the degree of seizure propagation or differences in neural substrates. Thus, the dataset provides a broad spectrum of seizure characteristics that reflect the inherent complexity of epileptic networks, emphasizing the need for individualized assessments when interpreting seizure-related electrophysiological data.

### Lack of spatial dependency in signal correlations

Figure 3B (left) displays the Euclidean distances between the MNI coordinates of the arrays for each patient and a representative iEEG electrode exhibiting the most epileptic activity (located in the PEZ where identifiable). We observe that, with the exception of patient 4, the other arrays are not located in close proximity to these electrodes. Figure 3B (right) illustrates the strength of the Pearson correlation between the broadband signal (in an epoch just before and during the early seizure) of the array, the representative iEEG electrode with the most epileptic activity, and the iEEG electrode closest to the Utah array. This plot indicates that spatial proximity does not lead to increased correlation between signals, as we do not observe a high correlation between the array and the closest iEEG contact for any of the patients. Instead, patients 1 and 4 demonstrate increased correlation between the iEEG contact nearest to the array and the iEEG contact exhibiting epileptic activity. Patients 2 and 4 show no correlation for any of the combinations, while patient 5 exhibits similarly low correlation for all three combinations.

### Increased number of ictal HFOs in contacts in the SOZ

**Fig. 4:**
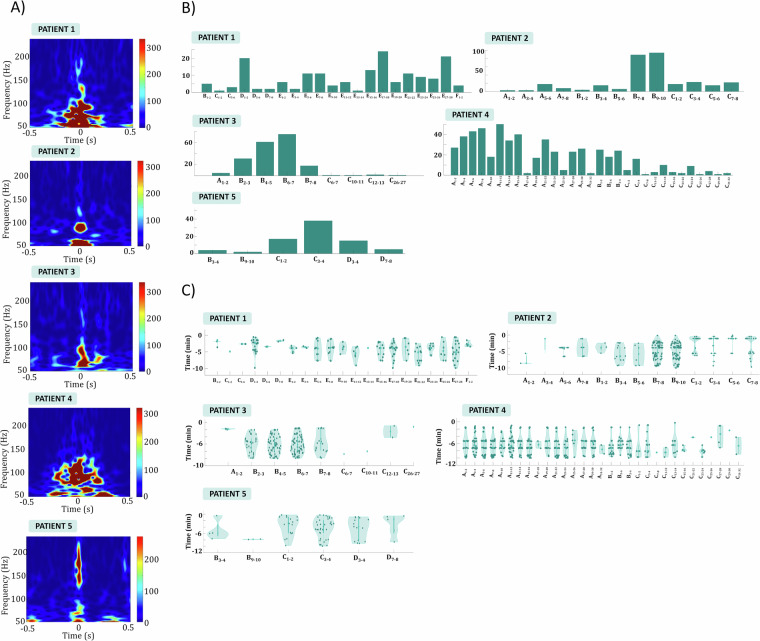
Ictal HFOs in the iEEG data. (**A**) Power spectrum of example HFOs detected in a representative channel for all patients. While distinct ictal HFO patterns are observed between patients, all exhibit clear HFO characteristics with frequencies ranging from 50 to 250 Hz. (**B**) The number of ictal HFOs detected throughout the entire epoch before seizure onset, depicted for all channels and patients. In all patients, contacts displaying pronounced epileptic activity also exhibit a significant number of ictal HFOs. C) Violin plots illustrating the distribution of time points at which ictal HFOs were detected. For patients 1, 2, 3, and 5, ictal HFOs do not exhibit a specific temporal pattern but instead appear randomly before the seizure. In contrast, for patient 4, ictal HFOs appear synchronized at four distinct time instances approximately at −10 minutes, −7 minutes, −5 minutes, and −2 minutes relative to seizure onset.

**Fig. 5:**
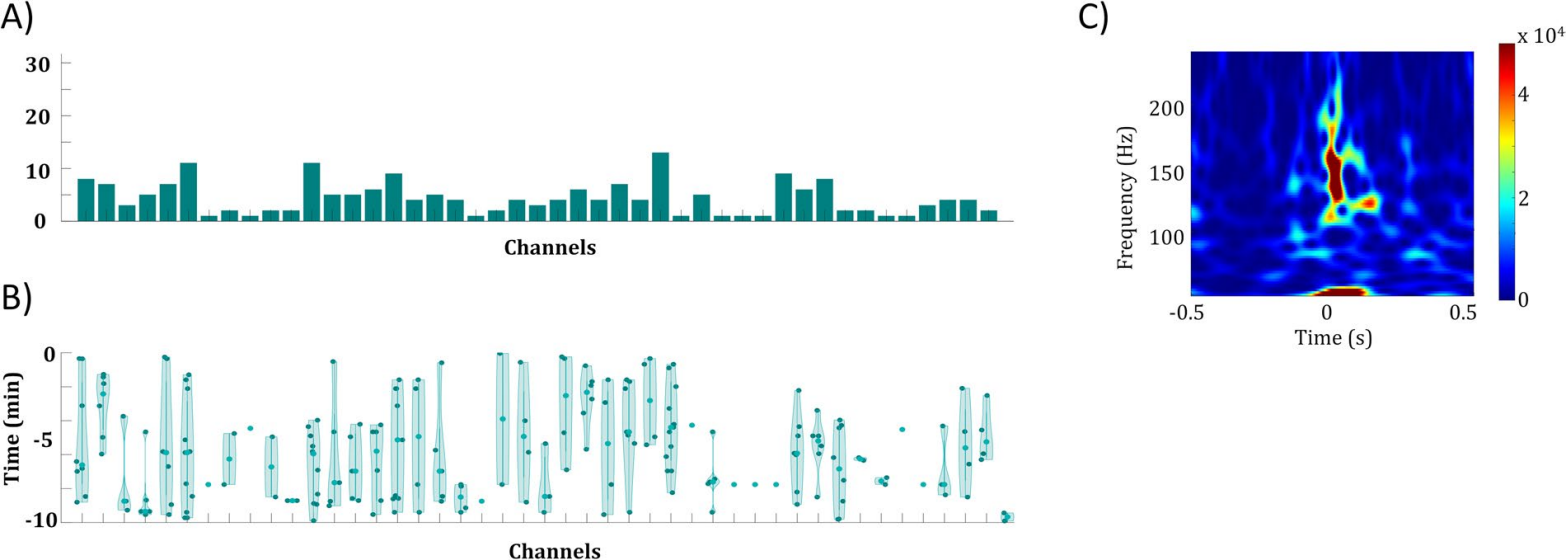
HFOs in the Utah array for Patient 5. (**A**) Number of ictal HFOs detected across all channels of the Utah array. While some channels show an increased number of ictal HFOs, the overall count remains relatively low. (**B**) Power spectrum of an example HFO detected in the Utah array. These ictal HFOs occur at higher frequencies (100 – 200 Hz) compared to the ictal HFOs observed in the iEEG data. (**C**) Violin plots illustrating the distribution of time points at which ictal HFOs were detected in the Utah array. Ictal HFOs do not exhibit a specific temporal pattern.

We assessed the ictal HFO quantity, timing, contact area, proximity to the SOZ, and their utility in identifying contacts with the most epileptic activity (Fig. 4). Notably, contacts with increased epileptic activity, excluding Patient 1, consistently exhibited an increased number of ictal HFOs before seizure onset (Fig. 4B). HFO timing patterns varied (Fig. 4C): Patients 2, 3, and 5 displayed a balanced distribution throughout the pre-seizure epoch, while Patient 4 showed distinct ictal HFO instances before seizures. In contrast, ictal HFOs were predominantly absent in the LFP signal from the Utah arrays, except for a few exceptions observed in Patient 5 (Fig. 5). In this case, certain ictal HFOs were indeed detected; however, they exhibited distinct characteristics, such as a shorter duration and a higher frequency range. These results indicate that the presence and temporal distribution of ictal HFOs are closely linked to the presumed epileptogenic zone, with a noticeable increase in the number of ictal HFOs prior to seizure onset in contacts showing heightened epileptic activity. The variability in HFO timing across patients suggests that different network mechanisms may govern the timing of ictal HFO generation relative to seizure onset. For example, the more uniform distribution of ictal HFOs in Patients 2, 3, and 5 may indicate sustained pre-seizure network hyperexcitability, while the distinct pre-seizure ictal HFO bursts in Patient 4 suggest more discrete, localized changes in excitability leading up to seizure onset. These findings support the use of ictal HFOs as potential biomarkers for identifying regions with increased epileptic activity, while also highlighting the need to consider individual differences in ictal HFO patterns for precise localization of the seizure onset zone.

### Significant MUA modulations preceding and following seizure onset

**Fig. 6:**
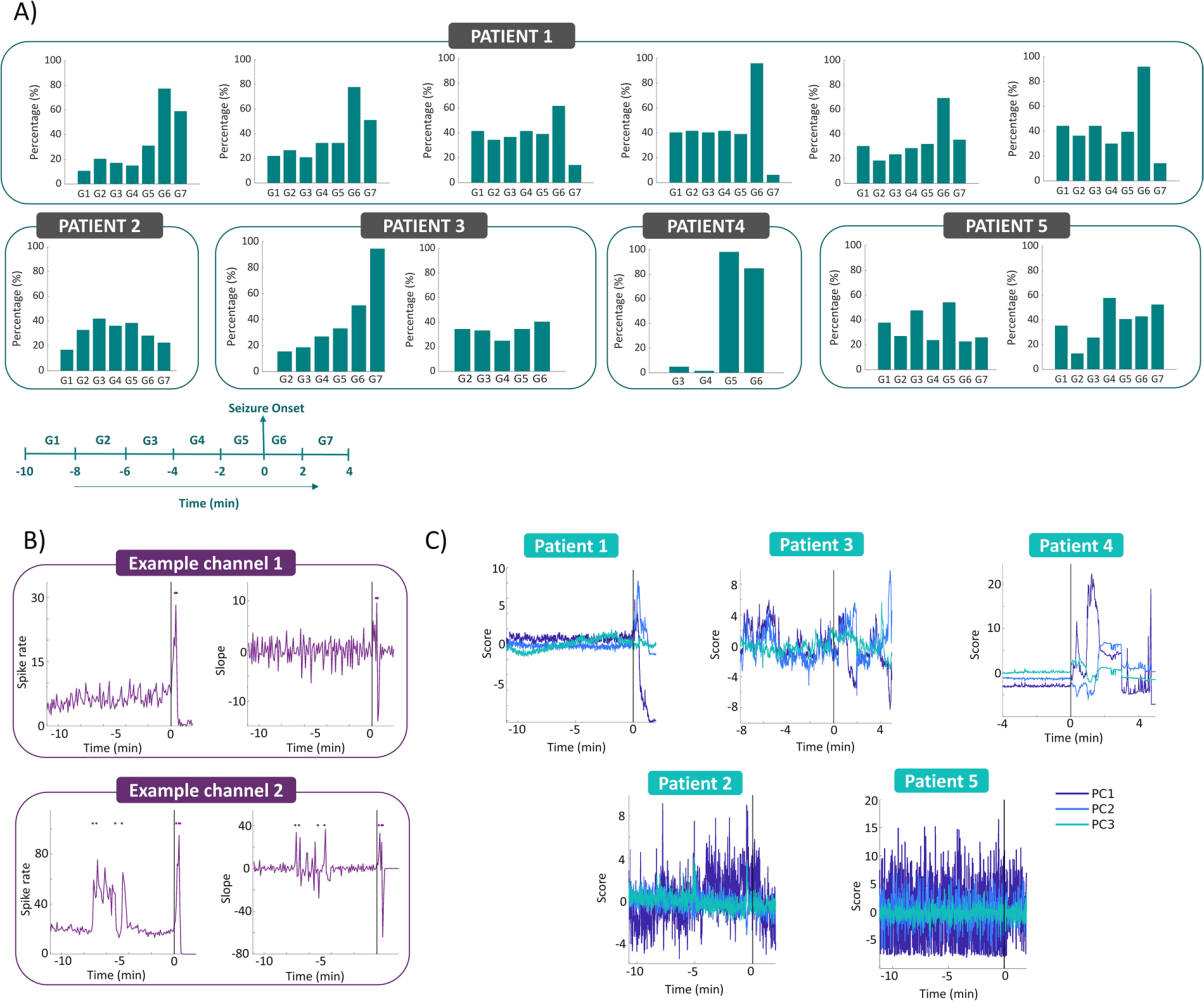
MUA modulations. (**A**) Barplots depicting the percentage of channels with significant modulations per time group for all seizures and patients. Except for patient 4, significant modulations are evident before seizure onset for all patients. (**B**) Illustration of how significant modulations were detected in two example channels. Channel 1 exhibits significant changes around seizure onset, while channel 2 shows changes before seizure onset. Spike rate and slope plots display these changes, with markers denoting time points exceeding 3 SD of the slope as significant modulations. (**C**) Principal Component Analysis (PCA) conducted on MUA activity across channels for each array. The first three principal components are plotted over time for one example seizure from all patients. Patients 1 and 4, those with less distinct seizure foci, display distinct modulations in PCs after seizure onset. For patient 3, with identified a presumed seizure focus and a Utah array outside the epileptogenic zone, post-seizure onset modulations are observable, primarily in PC1 and PC2, but less pronounced. In contrast, patients 2 and 5, both with identified presumed seizure onset zone and Utah arrays positioned outside the seizure onset zone, do not exhibit modulations in the PCs.

We categorized the time indices of significant modulations detected per channel into seven distinct groups, spanning from −10 minutes before the seizure to 4 minutes after, with a 2-minute interval. Subsequently, we computed the percentage of channels exhibiting significant modulations within each group. Figure 6A provides an overview of the percentage of channels displaying significant modulations across these defined time intervals for all seizures and patients. Figure 6B shows the spike rate and the slope for two example channels. For Patient 1, the group with the highest number of channels showing significant changes consistently spanned from the seizure onset until 2 minutes after seizure initiation across all six seizures. Additionally, seizures 1, 2, and 5 displayed a notable percentage of channels with significant modulations in the subsequent time interval (2 to 4 minutes after seizure onset), suggesting prolonged neural activity alterations following the seizure onset. Conversely, for seizures 3, 4, and 6, the percentage decreased after the seizure. Importantly, significant modulations were observed in a substantial number of channels even before seizure onset in all seizures. In contrast, for Patient 2, the time groups with the highest percentage of channels displaying significant modulations were observed before seizure onset, particularly from 6 minutes before the seizure until the seizure’s initiation. Patient 3 exhibited varying results for the two recorded seizures; the first seizure demonstrated a gradual increase in the percentage leading up to seizure onset, while the second seizure resembled the pattern seen in Patient 2, with consistent percentages of channels with significant modulations throughout the defined time groups. In the case of Patient 4, almost all modulations occurred after seizure onset, resulting in a notable disparity in percentages before and after the seizure. Finally, for Patient 5, significant modulations were observed across all time groups for seizure 1, albeit without a clear pattern. In seizure 3, the percentage peaked in the interval between −4 and −2 minutes before the seizure and remained relatively stable thereafter. In summary, our findings consistently identify significant modulations in neural activity, even preceding seizure onset, across all patients. However, it’s important to note that the specific patterns of these modulations are not consistent across different seizures or patients. This variability underscores the complexity of epileptic dynamics and highlights the need for individualized approaches in understanding epilepsy networks. The diverse patterns of MUA modulations observed across patients and seizures indicate that seizure-related neural activity can manifest differently depending on individual network configurations. The significant modulations detected before seizure onset, particularly in Patients 1 and 2, may suggest anticipatory changes in network excitability that could play a critical role in seizure generation. In contrast, the disparity noted in Patient 4, where modulations predominantly occurred post-onset, emphasizes the importance of temporal dynamics in the progression of seizure activity.

### PCA as a tool for tracking neural signal changes

We conducted PCA analysis across the channels and visualized the first three principal components (PCs) (Fig. 6C). Notably, for patients 1 and 4, where the seizure focus was less clear, we observed distinct modulations in PCs after seizure onset. In the case of patient 3, who had a well-defined seizure focus and a Utah array outside the epileptogenic zone, we also observed post-seizure onset modulations, albeit less pronounced and primarily confined to PC1 and PC2. Conversely, patients 2 and 5, who both had a clear seizure focus and Utah arrays positioned outside the seizure onset zone, did not exhibit any modulations in the PCs. This highlights the utility of PCA for capturing patterns of neural activity across channels, provided that the array is either within or in close proximity to areas with epileptic activity.

### Consistency of modulations across seizures

**Fig. 7:**
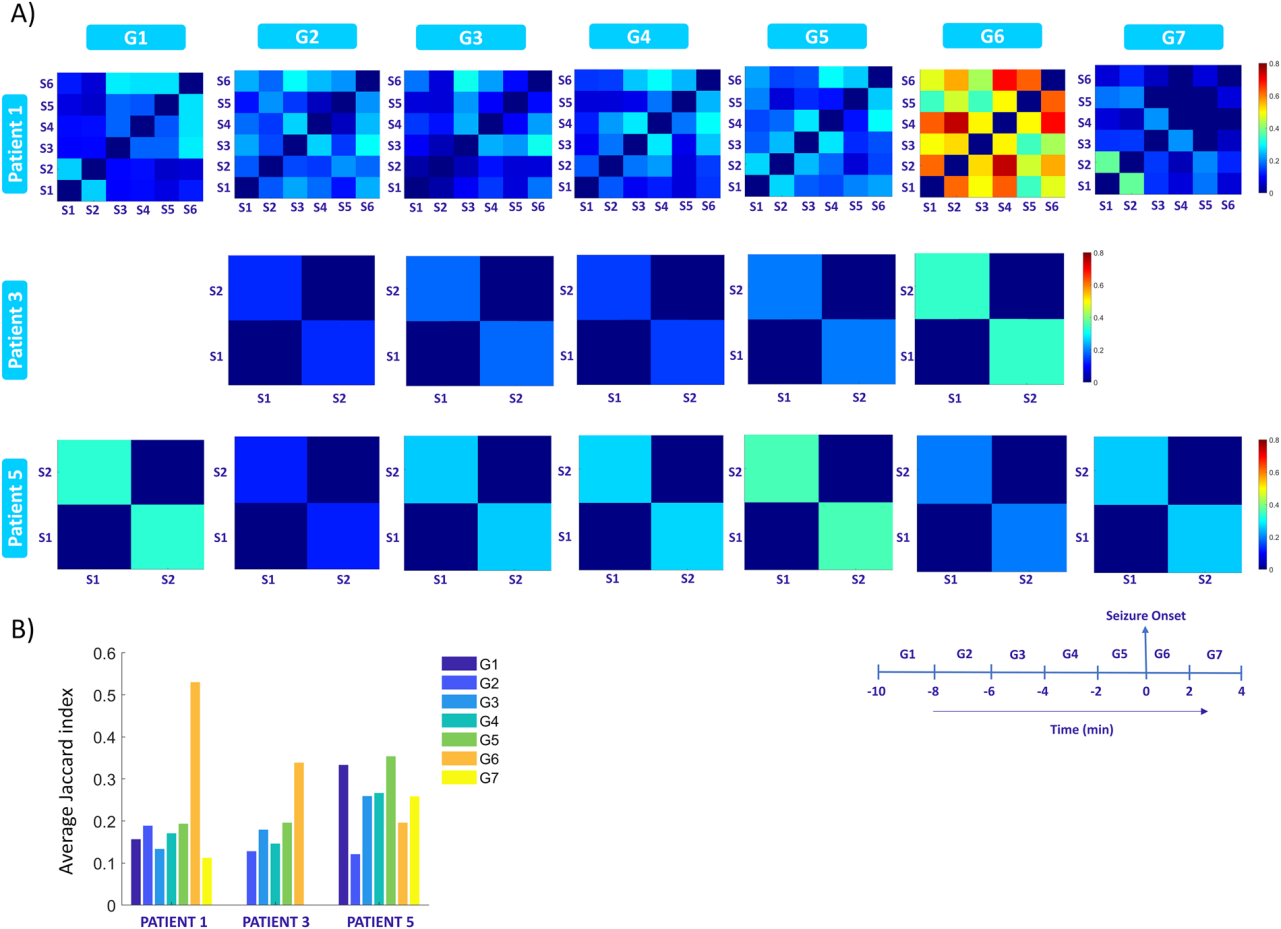
Consistency of MUA modulations across seizures. (**A**) Color plots show Jaccard coefficients between seizures for all time groups in three patients with multiple recorded seizures. Patient 1 had consistently low coefficients before seizure onset, spiking notably after seizure onset. Patient 3 showed an increasing trend leading up to the seizure and peaked 0 to 2 minutes after. Patient 5 displayed a less distinct pattern. (**B**) Barplots depict average Jaccard coefficients per time group, reaffirming the observed patterns.

In cases where multiple seizures were recorded, namely patients 1, 3, and 5, we assessed the consistency of channels exhibiting significant modulations within each time group across different seizures. The Jaccard coefficient served as our metric for comparing channels with significant modulations between all pairs of seizures, analyzed separately for each time group. Figure 7A graphically presents the coefficients for each time group across all patients, while Fig. 7B displays the average coefficient matrix encompassing all patients and time groups. Our findings revealed distinct patterns for each patient. For patient 1, the average Jaccard coefficient remained relatively low before seizure onset but exhibited a notable increase (more than doubling) during the time interval from seizure onset to 2 minutes thereafter. This suggests that channels with significant modulations began to exhibit consistency across seizures only in very close proximity to or after seizure onset. In the subsequent time group (2 to 4 minutes after seizure onset), the Jaccard index decreased, even falling below the pre-seizure level, indicating that channels displayed consistency primarily within the 0 to 2-minute window after seizure initiation. Patient 3 showed an increase in the Jaccard index leading up to the seizure onset. Similar to patient 1, patient 3 exhibited a peak in the 0 to 2-minute interval after seizure onset. Conversely, for patient 5, no distinct pattern emerged. The Jaccard index peaked in the interval −2 to 0 minutes before seizure onset but remained considerably lower compared to the peaks observed in patients 1 and 3. Overall, our analysis reveals that channels with significant modulations are consistently observed across all patients even before seizure onset, although the specific patterns of modulations vary across seizures and patients. Variability in the consistency of modulations among patients emphasizes the complexity of seizure dynamics. Patients 1 and 3 exhibited stronger synchronization in their modulating channels during the peri ictal period, while Patient 5’s lack of consistent patterns suggests a more heterogeneous network response. Understanding these differences can provide valuable insights into individual seizure mechanisms.

### Spike – local field potential (LFP) correlation

**Fig. 8:**
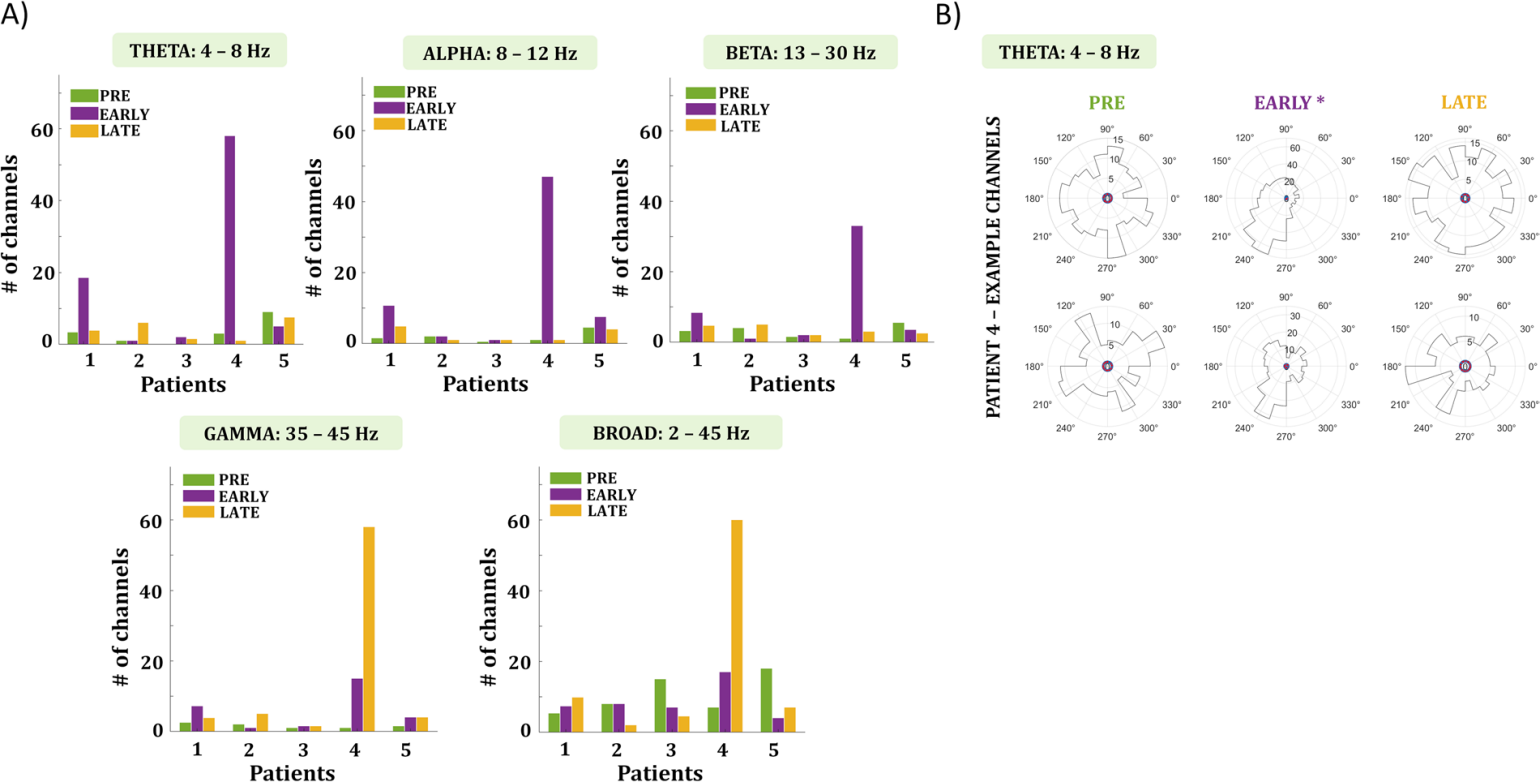
Spike–Field Coupling. (**A**) Bar plots indicate the number of channels with significant spike–field coupling for each frequency band (theta, alpha, beta, gamma, broadband). The height of each bar represents the number of channels with significant spike–field coupling per patient, while the color indicates the corresponding 5-second time interval (green: 5 minutes before the seizure [‘pre’], purple: immediately after seizure onset [‘early’], orange: 2 minutes after seizure onset [‘late’]). For the low-frequency bands (theta, alpha, beta), patient 4 shows coupling in multiple channels during the early seizure period. In contrast, for the higher frequencies (gamma) and the broadband signal, coupling appears to occur in the late seizure phase for patient 4. (**B**) Polar plots illustrate the phase coupling for two example channels of patient 4 across the three time segments in the theta band. In the pre and late segments, the distribution of spike times seems random; however, during the early period, firing is significantly coupled to a particular phase (~240°) of the LFP signal.

We evaluated the number of channels with significant spike–field coupling across five frequency bands and for all three time segments (Fig. 8A). For Patient 4, who did not have a clear seizure focus, exhibited more extensive seizure propagation, and had the Utah array placed closest to the presumed epileptogenic zone (PEZ), we observed numerous channels showing strong coupling during the ‘early’ period, but not during the ‘pre’ and ‘late’ periods (Fig. 8A,B). Consistent with the findings of Schevon *et al*.^12^, who demonstrated strong spike–phase coupling in the post-recruitment period using a similar approach, we observed strong coupling in more than half of the Utah array channels during the early period of the seizure for the theta frequency band. This phenomenon was also evident in the alpha and beta bands, albeit in fewer channels. Conversely, in the low gamma and broadband signals, this coupling was present in the majority of channels during the ‘late’ seizure period. This enhanced phase locking immediately following seizure onset may indicate a synchronized burst of activity involving multiple cortical and subcortical regions, facilitating widespread engagement of neural networks. The findings in Patient 4 align with those previously reported in Schevon *et al*.^12^, which is noteworthy given that Patient 4 had electrode placement within or in close proximity to the PEZ, similar to the patients in their study. In contrast, the MEAs in our other patients, except for Patient 4, were positioned in areas distant from the PEZ. This suggests that the observed coupling may be an intrinsic property of the epileptogenic zone.

## Usage Notes

The provided data with this manuscript can be easily imported into MATLAB, and custom-written scripts, EEGLAB, or any other signal processing toolbox can be utilized for visualization and further processing. The data have already been cleaned and aligned to the onset of the seizure, making it straightforward to perform additional analyses. The inclusion of the broadband signal for both LFP and iEEG data is particularly advantageous as it enables users to investigate various frequencies. Notably, the iEEG data are sampled at a high frequency (Fs = 1024) allowing the study of not only ictal HFOs, but also fast ripples (250–500 Hz).

Obtaining microarray (MEA) data from human subjects, particularly during seizure events, is an exceedingly rare occurrence. Consequently, this dataset represents an invaluable resource for gaining insights into the intricate mesoscale dynamics of epilepsy. It includes recordings of both Local Field Potentials (LFP) and Multi-Unit Activity (MUA) obtained from Utah arrays placed at various distances from the Seizure Onset Zone (SOZ). The consistent proximity of the Utah arrays to a macroelectrode contact facilitates research on the relationships between iEEG and nearby MUA and high-gamma LFP activity, especially in the context of ictal high-frequency oscillations (HFOs).

The dataset also includes seizures, with data where a presumed seizure onset zone could be identified and from patients with unclear foci, offering a comprehensive view of epilepsy dynamics. While none of the patients became completely seizure-free, some experienced improvements following identification of the seizure onset zone. By combining both iEEG and Utah array data, researchers can explore a wide range of aspects, including connectivity at different scales, synchronization patterns, spatiotemporal phenomena like propagating waves, and cross-frequency couplings within the Utah arrays, either independently or in conjunction with macrocontacts and compare between seizure types.

Ultimately, this dataset provides valuable insights into micro, meso, and macro epileptic networks, advancing our understanding of the intricate neural processes behind epilepsy—an important step toward better seizure prediction and enhanced care for epilepsy patients.

## Data Availability

The code, along with instructions, for visualizing the raster plot of the MUA and computing/plotting power spectra of all channels for both LFP and SEEG (Fig. 2) is available in the Ebrains repository, provided together with the dataset^27^.

## References

[1] Zaccara, G., Franciotta, D. & Perucca, E. Idiosyncratic adverse reactions to antiepileptic drugs. *Epilepsia* **48**, 1223–1244 (2007). 17386054 10.1111/j.1528-1167.2007.01041.x

[2] Perucca, P., Carter, J., Vahle, V. & Gilliam, F. G. Adverse antiepileptic drug effects Toward a clinically and neurobiologically relevant taxonomy. (2009). 10.1212/01.wnl.0000345667.45642.61 PMC2677485 19349601

[3] Engel, J., Bragin, A., Staba, R. & Mody, I. High-frequency oscillations: What is normal and what is not? *Epilepsia* **50**, 598–604 (2009). 19055491 10.1111/j.1528-1167.2008.01917.x

[4] Zijlmans, M. *et al*. High-Frequency Oscillations as a New Biomarker in Epilepsy. 10.1002/ana.22548. 10.1002/ana.22548 PMC3754947 22367988

[5] Weiss, S. A. *et al*. Ictal high frequency oscillations distinguish two types of seizure territories in humans. *Brain* **136**, 3796–3808 (2013). 24176977 10.1093/brain/awt276 PMC3859220

[6] Weiss, S. A. *et al*. Seizure localization using ictal phase-locked high gamma: A retrospective surgical outcome study. *Neurology* **84**, 2320–2328 (2015). 25972493 10.1212/WNL.0000000000001656 PMC4464742

[7] Smith, E. H. *et al*. The ictal wavefront is the spatiotemporal source of discharges during spontaneous human seizures. *Nat. Commun*. **7** (2016). 10.1038/ncomms11098 PMC4820627 27020798

[8] Modur, P. N., Zhang, S. & Vitaz, T. W. Ictal high-frequency oscillations in neocortical epilepsy: implications for seizure localization and surgical resection. *Epilepsia* **52**, 1792–1801 (2011). 21762451 10.1111/j.1528-1167.2011.03165.x PMC3188690

[9] Jacobs, J. *et al*. High-Frequency Electroencephalographic Oscillations Correlate With Outcome of Epilepsy Surgery. *ANN NEUROL* **67**, 209–220 (2010). 20225281 10.1002/ana.21847 PMC3769290

[10] Ray, S. & Maunsell, J. H. R. Different Origins of Gamma Rhythm and High-Gamma Activity in Macaque Visual Cortex. *PLoS Biol* **9**, 1000610 (2011). 10.1371/journal.pbio.1000610 PMC3075230 21532743

[11] Schevon, C. A. *et al*. Microphysiology of epileptiform activity in human neocortex. *J. Clin. Neurophysiol.* **25**, 321–330 (2008). 18997628 10.1097/WNP.0b013e31818e8010 PMC2967462

[12] Schevon, C. A. *et al*. Evidence of an inhibitory restraint of seizure activity in humans. *Nat. Commun. 2012 31* **3**, 1–11 (2012). 10.1038/ncomms2056 PMC3658011 22968706

[13] Bower, M. R., Stead, M., Meyer, F. B., Marsh, W. R. & Worrell, G. A. Spatiotemporal neuronal correlates of seizure generation in focal epilepsy. *Epilepsia* **53**, 807–816 (2012). 22352423 10.1111/j.1528-1167.2012.03417.x PMC3339564

[14] Wyler, A. R., Ojemann, G. A. & Ward, A. A. Neurons in human epileptic cortex: correlation between unit and EEG activity. *Ann. Neurol.* **11**, 301–308 (1982). 7092182 10.1002/ana.410110311

[15] Isokawa-Akesson, M., Wilson, C. L. & Babb, T. L. Structurally stable burst and synchronized firing in human amygdala neurons: Auto- and cross-correlation analyses in temporal lobe epilepsy. *Epilepsy Res.* **1**, 17–34 (1987). 3504380 10.1016/0920-1211(87)90047-7

[16] Truccolo, W. *et al*. Single-neuron dynamics in human focal epilepsy. *Nat. Neurosci. 2011 145* **14**, 635–641 (2011). 10.1038/nn.2782 PMC3134302 21441925

[17] Truccolo, W. *et al*. Neuronal Ensemble Synchrony during Human Focal Seizures. *J. Neurosci.* **34**, 9927–9944 (2014). 25057195 10.1523/JNEUROSCI.4567-13.2014 PMC4107409

[18] Schevon, C. A. *et al*. Multiscale recordings reveal the dynamic spatial structure of human seizures. *Neurobiol. Dis.* **127**, 303–311 (2019). 30898669 10.1016/j.nbd.2019.03.015 PMC6588430

[19] Truccolo, W. *et al*. Single-neuron dynamics in human focal epilepsy. *Nat. Neurosci.* **14**, 635–643 (2011). 21441925 10.1038/nn.2782 PMC3134302

[20] Park, Y. S. *et al*. Early Detection of Human Epileptic Seizures Based on Intracortical Microelectrode Array Signals. *IEEE Trans. Biomed. Eng.* **67**, 817–831 (2020). 31180831 10.1109/TBME.2019.2921448 PMC7067044

[21] Bougou, V. *et al*. Neuronal tuning and population representations of shape and category in human visual cortex. *Nat. Commun. 2024 151* **15**, 1–15 (2024). 10.1038/s41467-024-49078-3 PMC11139926 38816391

[22] Ramirez, J. G. *et al*. Intracortical recordings reveal the neuronal selectivity for bodies and body parts in the human visual cortex. *Proc. Natl. Acad. Sci*. **121** (2024). 10.1073/pnas.2408871121 PMC11665852 39652751

[23] Xia, M., Wang, J. & He, Y. BrainNet Viewer: A Network Visualization Tool for Human Brain Connectomics. *PLoS One* **8**, 68910 (2013). 10.1371/journal.pone.0068910 PMC3701683 23861951

[24] Blenkmann, A. O. *et al*. Ielectrodes: A comprehensive open-source toolbox for depth and subdural grid electrode localization. *Front. Neuroinform.* **11**, 14 (2017). 28303098 10.3389/fninf.2017.00014 PMC5333374

[25] Delorme, A. & Makeig, S. EEGLAB: An open source toolbox for analysis of single-trial EEG dynamics including independent component analysis. *J. Neurosci. Methods* **134**, 9–21 (2004). 15102499 10.1016/j.jneumeth.2003.10.009

[26] de Cheveigné, A. ZapLine: A simple and effective method to remove power line artifacts. *Neuroimage* **207** (2020). 10.1016/j.neuroimage.2019.116356 31786167

[27] Bougou, V. *et al*. Mesoscale Insights in Epileptic Networks: A Multimodal Intracranial Dataset (v2) [Data set]. *EBRAINS.*10.25493/ZC9M-2ES (2025). 10.1038/s41597-025-05026-4 PMC12065801 40348768

[28] Zhou, G. *et al*. Novel Tools and Methods HFOApp: A MATLAB Graphical User Interface for High-Frequency Oscillation Marking. 10.1523/ENEURO.0509-20.2021 (2021). 10.1523/ENEURO.0509-20.2021 PMC8503963 34544760

[29] Benoît, B. *et al*. Mapping interictal oscillations greater than 200 Hz recorded with intracranial macroelectrodes in human epilepsy. *A J. Neurol*. 10.1093/brain/awp277. 10.1093/brain/awp277 19920064

